# Precipitating Factors for Functional Cognitive Disorder

**DOI:** 10.1111/ene.70082

**Published:** 2025-03-04

**Authors:** Veronica Cabreira, Laura McWhirter, Jon Stone, Alan Carson

**Affiliations:** ^1^ Centre for Clinical Brain Sciences University of Edinburgh Edinburgh UK

**Keywords:** brain injury, cognitive disorders, COVID‐19, functional cognitive disorder, functional neurological disorder, migraine, precipitating factors, life event

## Abstract

**Background:**

The underlying mechanisms of functional cognitive disorder (FCD) are still poorly understood. This hinders diagnostic identification and establishment of personalised and effective treatments. We aimed to describe the precipitating factors for new‐onset FCD and explore differences between the acute and gradual onset FCD groups.

**Methods:**

Retrospective analysis of a consecutive sample of FCD patients seen in three dedicated clinics in the United Kingdom between September 2023 and March 2024. Only patients with at least one‐year symptom duration were included. We extracted mode of onset and precipitating factors, demographics, comorbidities, family history of cognitive symptoms, personal exposure to dementia, symptom duration and trajectory, as well as cognitive testing when available.

**Results:**

Ninety‐three patients were included, of which 45 had an acute onset of functional cognitive symptoms (48% of total). Of the acute onset group, 51% were female; mean age of symptom onset was 44 years (± 12). In the acute onset group, COVID‐19 (*n* = 18, 40%), head injury (*n* = 16, 36%) and migraine (*n* = 16, 36%) were the most common precipitating factors for symptom onset, followed by cardiovascular/vasovagal events, dissociative episodes, panic, medication change, medical procedure under sedation and acute vertigo. The acute onset group was younger, most commonly had a fluctuating course of symptoms, and more headache and fatigue than the gradual onset group.

**Conclusions:**

Functional cognitive disorder often begins acutely. The circumstances around onset may have treatment and prognostic implications. Similar to other functional disorders, the experience of abnormal cognition from an acute pathophysiological event can act as powerful precipitating factors for functional cognitive disorder.

## 1 Introduction

Functional cognitive disorder (FCD) is the cognitive subtype of functional neurological disorder (FND) [1]. The term is used to describe cognitive symptoms that are persistent and distressing and whose distinguishing feature is the presence of *internal inconsistency* [2]. FCD accounts for up to a quarter of patients attending memory services [3] and is also common in other medical conditions such as chronic fatigue syndrome, fibromyalgia and irritable bowel syndrome [4].

Work in the last 10–15 years has highlighted the importance of acute precipitating factors in functional disorders, which may help explain the nature of the symptom. For example, functional motor symptoms may be precipitated by an injury in the relevant limb [5, 6], or persistent postural perceptual dizziness (PPPD) is usually triggered by an acute experience of dizziness occurring for another reason [7]. Much less is known about specific risk factors and mechanisms responsible for the development of FCD. Understanding the relationship between functional cognitive symptoms and acute precipitating factors may provide further clues regarding specific triggers and potential underlying mechanisms for FCD.

Therefore, in this study, we sought to characterise the circumstances of symptom onset in FCD patients and investigate whether the acute group exhibits different characteristics from the gradual onset group, by analysing mode of onset, precipitating factors, demographics, comorbidities, family history of cognitive symptoms, personal exposure to cognitive difficulties, symptom duration and trajectory, and cognitive performance.

## 2 Methods

### 2.1 Patient Selection

As part of a larger FCD diagnostic study [8], data from consecutive patients with an FCD diagnosis attending three dedicated clinics (two neuropsychiatrists and one senior neurology trainee with an interest in cognition), in NHS Lothian, Scotland, between September 2023 and March 2024, were retrospectively extracted from medical electronic records.

The diagnosis of FCD was made if patients fulfilled the diagnostic criteria published in 2020 (2): (1) one or more symptoms of impaired cognitive function; (2) evidence of internal inconsistency; (3) symptoms or deficit not better explained by another medical or psychiatric disorder; and (4) symptoms causing significant distress or impairment in social or other important areas of functioning or warrants medical evaluation. All patients had at least 12 months of follow‐up for diagnostic confirmation. Patients with an uncertain diagnosis and ongoing investigations were excluded. All evaluating clinicians had expertise in the diagnosis of FND and cognitive symptoms and were previously involved in diagnostic and expert consensus studies for FCD [3, 8, 9]. The diagnosis of FCD was supported by investigations in a pragmatic manner according to current practices and local standards [8].

### 2.2 Data Collection

As part of routine assessment interviews, patients were asked about the mode of onset and precipitating factors (in the acute group) of cognitive symptoms, demographics, past or present comorbidities, family history of cognitive symptoms, occupational exposure to cognitive difficulties, symptom duration and trajectory. A cognitive screening test (Addenbrooke's cognitive examination III) was performed when deemed appropriate by the direct care team. The acute onset group was defined as cognitive symptoms starting within hours or days post initial trigger. The deidentified data in clinical letters was extracted using a standardised chart review for all patients.

### 2.3 Statistical Analysis

The frequency of individual precipitating events at onset in the acute group was analysed. Patient characteristics were compared between the two groups (acute onset vs. gradual onset FCD) using Mann–Whitney *U*‐tests for continuous variables and Fisher exact tests for categorical variables. A threshold of *p* < 0.05 indicates statistical significance. All analyses were performed using R (version 4.3.1). Ethics approval was obtained from the University of Edinburgh (23‐EMREC‐026).

## 3 Results

A total of 93 patients with FCD were identified, of whom 45 (48%) had an acute onset of symptoms and 48 (52%) had a gradual symptom progression. Patients with an acute onset of symptoms had an onset of cognitive symptoms at a younger age than the gradual group (44 (±12) versus 52 (±14) years‐old, *p* = 0.007). Of the acute onset group, 23 (51%) were female, and mean symptom duration was 2.5 years (±2) (Table 1).

**TABLE 1:** Demographics and comorbidities according to mode of onset of cognitive symptoms (acute vs. gradual onset).

	Acute onset (*n* = 45)	Gradual onset (*n* = 48)	Total (*n* = 93)	*p*
	*n* (%)	*n* (%)	*n* (%)	
Age at symptom onset (mean (SD))	44.2 (11.8)	51.7 (13.9)	48.1 (13.4)	**0.007**
Age at diagnosis (mean (SD))	46.7 (11.9)	56.7 (8.7)	51.9 (11.5)	**< 0.001**
Gender = male	22 (48.9)	23 (47.9)	45 (48.4)	1.000
History of head injury	16 (35.6)	3 (6.2)	19 (20.4)	**0.001**
Before the FCD diagnosis	0	3 (6.2)		
Concomitant/post‐FCD diagnosis	16 (35.6)	0		
Psychological stressors around symptom onset	31 (68.9)	36 (75.0)	67 (72.0)	0.671
Diagnosis of anxiety/depression	20 (44.4)	21 (43.8)	41 (44.1)	1.000
PTSD	4 (8.9)	4 (8.3)	8 (8.6)	1.000
Neurodevelopmental disorder (ASD/ADHD)	3 (6.7)	1 (2.1)	4 (4.3)	0.564
Sleep problems non‐OSA	5 (11.1)	5 (10.4)	10 (10.8)	1.000
Obstructive sleep apnoea	1 (2.2)	7 (14.6)	8 (8.6)	0.079
Migraine and other headaches	16 (35.6)	4 (8.3)	20 (21.5)	**0.003**
Chronic pain (non‐migraine)	2 (4.4)	4 (8.3)	6 (6.5)	0.733
Fatigue	14 (31.1)	2 (4.2)	16 (17.2)	**0.002**
Dizziness with PPPD features	5 (11.1)	2 (4.2)	7 (7.5)	0.381
Dissociative symptoms (depersonalisation/derealisation)	4 (8.9)	5 (10.4)	9 (9.7)	1.000
Other FNDs (motor/seizures/sensory)	8 (17.8)	11 (22.9)	19 (20.4)	0.721
Before the FCD diagnosis	3 (6.7)	0		
Concomitant/post‐FCD diagnosis	5 (11.1)	11 (22.9)		
Inflammatory bowel syndrome	3 (6.7)	2 (4.2)	5 (5.4)	0.941
Perimenopause symptoms	4 (8.9, 17% of female patients)	3 (6.2, 12% of the female patients)	7 (7.5)	0.929
Other medical comorbidities^a^	8 (17.8)	11 (22.9)	19 (20.4)	0.721
Cognitive performance (ACE‐III)				1.000
Normal performance (score within normative range)	35 (77.8)	38 (79.2)	73 (78.5)	
Missing	9 (20)	5 (10.4)	14 (15)	
Family history of dementia	10 (22.2)	13 (27.1)	23 (24.7)	0.762
Working as a carer	16 (35.6)	8 (16.7)	24 (25.8)	0.065
Distractible and/or fluctuating symptoms	40 (88.9)	32 (66.7)	72 (77.4)	**0.021**
Stable or improving over time	43 (95.6)	44 (91.7)	87 (93.5)	0.733

*Note*: Bold: *p* < 0.05.

Abbreviations: ACE‐III, Addenbrooke's cognitive examination III; ADHD, attention‐deficit/hyperactivity disorder; ASD, autism spectrum disorder; FND, functional neurological disorder; OSA, obstructive sleep apnoea; PPPD, persistent postural perceptual dizziness; PTSD, post‐traumatic stress disorder.

^a^ Medical comorbidities included cancer (*n* = 6), asthma (*n* = 2), rheumatoid arthritis (*n* = 2), severe hearing loss (*n* = 2), giant cell arthritis (*n* = 1), primary biliary cholangitis (*n* = 1), brain white‐matter lesions (suspicion of multiple sclerosis) (*n* = 1), stroke (*n* = 1), REM sleep behaviour disorder (*n* = 1), antiphospholipid syndrome (*n* = 1), small fibre neuropathy (*n* = 1), and ulcerative colitis (*n* = 1).

### 3.1 Circumstances at Onset in the ‘Acute’ Group

In the acute onset group, COVID‐19 infection (*n* = 18, 40%, none with delirium), probable or symptomatic possible mild traumatic brain injury (Mayo Head Injury Classification System [10]) (*n* = 16, 36%), migraine attack or another severe headache episode (*n* = 16, 36%; of these, four did not have a prior diagnosis of migraine but presented with a new‐onset severe headache episode with migraine‐like characteristics), cardiovascular (myocardial infarction and cardiac ablation for ventricular tachycardia) and vasovagal events (*n* = 3, 7%), and a dissociative episode (*n* = 3, 7%) were the most common events identified as precipitating factors for new onset of cognitive symptoms. Other events happening in one patient each were as follows: a panic attack, recent medication change for primary biliary cholangitis, a nonsurgical medical procedure under deep sedation (endoscopic retrograde cholangiopancreatography) and an acute episode of peripheral vertigo.

**FIGURE 1:**
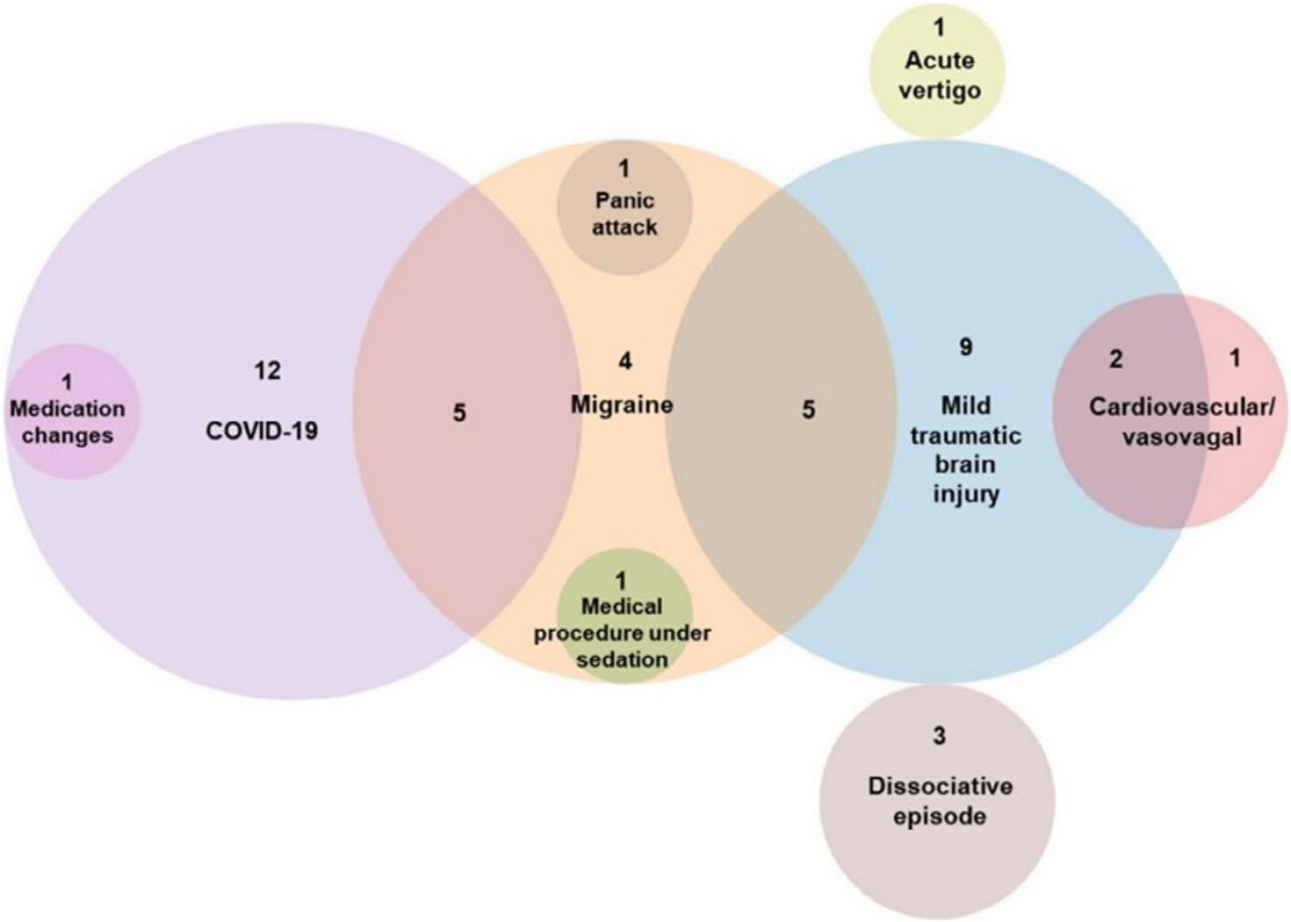
Venn diagram showing the frequency of precipitating factors/triggers at symptom onset, and their overlap, in the acute onset group. All patients in the acute onset group had an identifiable precipitating factor.

Fifteen of the acute onset patients had two concomitant events associated with cognitive symptom onset, and the two most common associations were mild traumatic brain injury with migraine (common as part of a ‘post‐concussion syndrome'), and migraine with COVID‐19 infection (Figure 1).

### 3.2 Comorbidities and Disease Trajectory

Having a history of head injury, migraine and fatigue either before or after a precipitating event was more common in the acute onset group. Other concomitant symptoms and comorbidities were not different between groups. Twenty patients (44%) had received a formal diagnosis of a mood disorder (anxiety and depression), either in the past or post‐acute event. In the acute onset group, 10 patients (22%) had a family history of cognitive symptoms, seven of whom had onset at an older age (> 65 years of age). Sixteen patients (36%) currently or previously worked as carers or in mental health services. Of the 89 (85%) patients with cognitive performance data, the proportion of patients who scored within normative range was similar in the acute and gradual onset groups (79% of the total cohort), with attention being the most affected domain.

Cognitive symptoms with an acute onset more often followed a fluctuating course (89% vs. 67% in the gradual onset group, *p* = 0.021). In 94% of our cohort, symptoms remained stable or somewhat improved since the onset, while the remaining patients continued to worsen after the initial event; there was no difference between groups, although the absence of improvement was numerically more common in the gradual onset group and driven by comorbid PTSD in the acute onset group (Table 1).

## 4 Discussion

The present study identified that functional cognitive symptoms began acutely in about half of the patients in our FCD cohort, with nine different acute precipitants.

In line with previous studies including seizure and motor FND phenotypes, we found that injury at onset, in this case head injury, can precipitante FCD [5, 6, 11, 12, 13]. Just as limb weakness and immobilisation is an integral and natural component response to limb injury, initial cognitive symptoms are an integral and directly attributable feature of mild traumatic brain injury. However, FCD involves symptoms that persist beyond the expected window of recovery for mild traumatic brain injury (initial weeks to months) [11, 14, 15], and take on a quality, with internal inconsistency, that would not be expected in acute brain injury. In previous reports, cognitive symptoms persisting longer than expected after mild traumatic brain injury and other forms of physical injury correlated poorly with performance on neuropsychological testing and even the presence of brain injury [16, 17]. Instead, perceptions about the cause and outcome of symptoms, pre‐injury depressive or anxiety disorder, and acute post‐traumatic stress were better predictors of persistence cognitive symptoms [17, 18].

Migrainous cognitive symptoms, and likely alarming acute pain, may also alter brain predictions in a way that makes cognitive symptoms persistent. Over 80% of migraine sufferers report cognitive difficulties including brain fog, confusion and slow thinking before, during and after a migraine attack [19, 20]. Migraine patients may also be more vulnerable to cognitive symptoms when exposed to additional triggers such as a head injury or COVID‐19 infection, and vice‐versa. Taken together, this suggests that migraine may act as both a potential predisposing and precipitating factor for FCD, and these findings are supported by reports of a correlate between the location of a precipitating physical injury and the subsequent functional symptom across various functional disorders [21], an aspect that can be considered in future educational and therapeutic strategies.

Eighteen patients in our cohort fulfilled diagnostic criteria for FCD after COVID‐19 infection. Further research is needed to unravel the likely multiple causes of cognitive symptoms in long‐COVID [22]. These data does not suggest that all cognitive symptoms as part of long‐COVID relate to FCD. However, a range of research findings may be in keeping with this being the case in a subset of patients. A detailed analysis of the most affected cognitive domains in long‐COVID patients reveals that working memory, attention and concentration difficulties tend to dominate the picture [23], resembling other FCDs and functional cognitive symptoms present in other functional disorders [4]. Current evidence also supports a poor correlation between perceived cognitive impairment and objectively measured cognitive performance in post‐COVID‐19 infection [23, 24, 25], with cognitive symptoms independent from the severity of the initial COVID infection [26], and mostly driven by fatigue and depression [23, 25]. An area of further enquiry is the potential suboptimal task engagement and task effort found at higher rates in long‐COVID patients in comparison with other neurological disorders [26, 27, 28]. Authors from a systematic review on long‐COVID studies found similarities between manifestations of long‐COVID and the cluster of symptoms observed in functional disorders although data were insufficient to support or refuse a diagnosis of FND based on positive features [29]. Importantly, in long‐COVID series, cognitive problems, including memory difficulties, fatigue, headache and sleep disorders, were the most commonly observed symptoms, as in our study (Box 1). It is likely that some evolve to a full FCD phenotype over time, and positive features supporting this diagnosis should be explored, which may open complementary treatment avenues.

BOX 1 **Illustrative case.**

A 54‐year‐old lady with a history of primary biliary cholangitis and generalised anxiety disorder controlled with medication, attended our service complaining of cognitive and sensory symptoms. She works as a carer for her daughter. Eighteen months prior, she had experienced a mild COVID‐19 infection and did not need to be admitted in the hospital. Around the same time, her medication for her chronic condition was changed with the introduction of ursodeoxycholic acid. Within days, she started experiencing constant fatigue, breathlessness, concentration and word‐finding difficulties, frequent dissociation, and sleep disturbance. She had never experienced cognitive difficulties prior to this infection. The patient was concerned over an ensuing damage to her brain caused by COVID‐19 and her primary condition. She gave a detailed report of her cognitive difficulties and her day‐to‐day life, which included managing the house, finances, driving and reading difficulties. She recalled previous interactions with other healthcare professionals and dates of different memory lapse episodes. A link between her cognitive symptoms and her fatigue levels was established, and she reported some daily fluctuation. On a cognitive screening test (Addenbrooke's cognitive examination‐III), she scored within the normative range. An MRI done prior to this assessment was also unremarkable. A diagnosis of functional cognitive disorder was made, and an individual formulation discussed with the patient. In addition, she was offered cognitive behavioural therapy for her cognitive symptoms and introduced to the principles of pacing strategies and sleep hygiene. At 12‐week follow‐up, she reported significant improvement in cognitive symptoms and dissociation that she attributed to a better understanding of her condition, reduced concern about brain damage and better management of her stress levels with breathing exercises.

A range of other precipitating events for FCD in our cohort included cardiovascular/vasovagal episodes, dissociative events, panic, medication changes, medical procedure under deep sedation and acute peripheral vertigo, all of which have been described for other functional disorders [5, 6, 30, 31].

In the majority of our cohort, symptoms remained stable or somewhat improved after an abrupt symptom onset with a minimum 12‐month follow‐up, albeit PTSD and other severe psychiatric comorbidities may predict a poorer prognosis. Individuals with acute onset functional cognitive symptoms had a similar profile to those with gradual symptom onset groups, with the exception of fatigue and migraine, suggesting that the mode of onset may not greatly influence the overall course of the condition.

**FIGURE 2:**
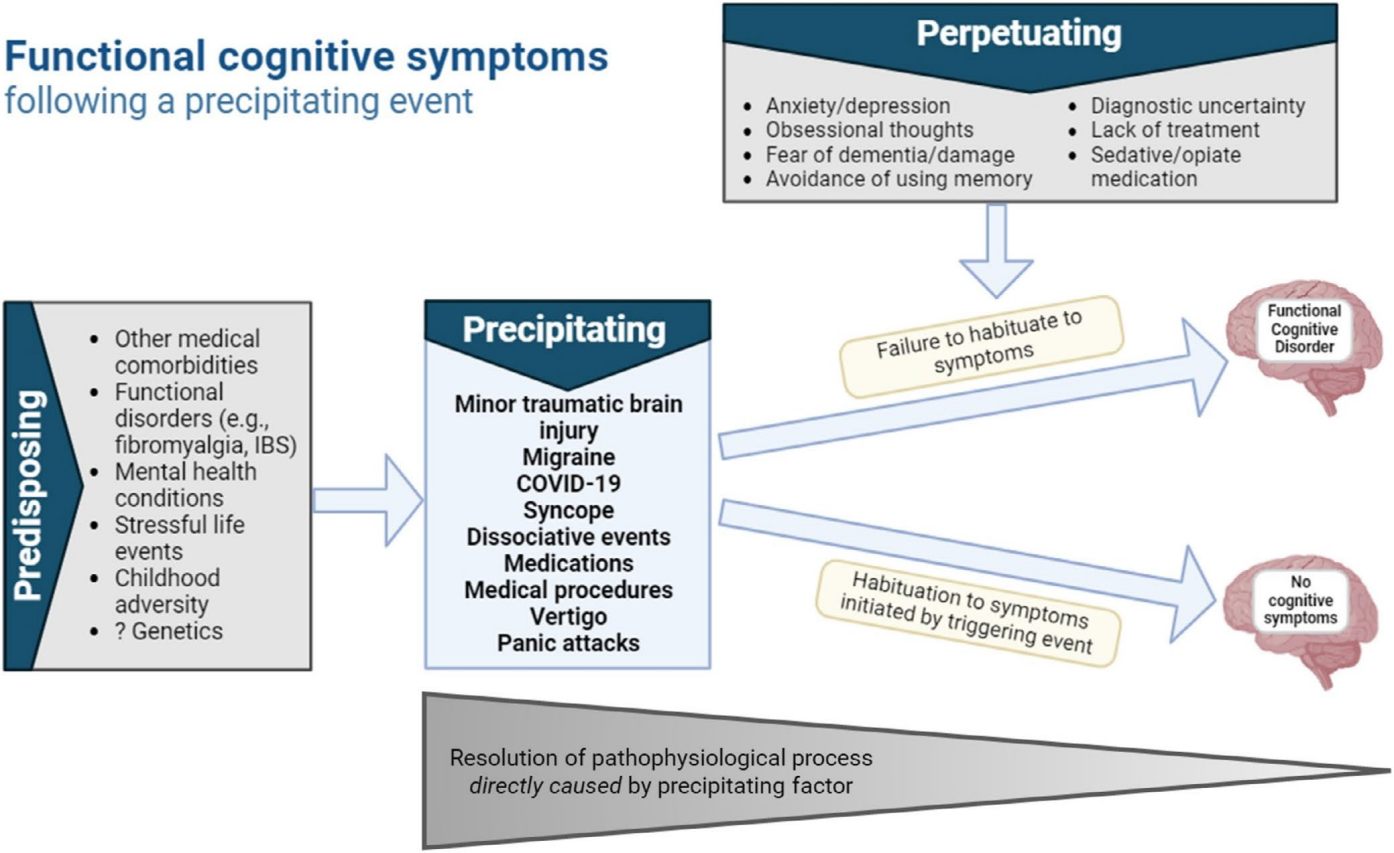
Symptom model for acute onset of functional cognitive disorder. Various precipitating factors work through potential direct effects, followed by central sensitisation, increased self‐monitoring, activation of a danger response with emotional, cognitive and behavioural changes, which drive further cognitive symptoms (i.e., failure of habituation to the acute trigger when the direct effects have improved). Precipitating factors may be directly relevant to mechanisms underlying FCD, or simply reveal an underlying predisposition to functional cognitive symptoms that is brought up by common triggers in already vulnerable individuals. IBS: inflammatory bowel syndrome.

The findings of the present study fit a model whereby the expectation and/or experience of abnormal cognition following different types of pathophysiological event, work through a range of potential common mechanisms to lead to symptom persistence including attentional dysregulation [4, 32]. Acute triggers, and their associated potential direct effects, update a ‘symptom internal model’ with new salient information that is interpreted as the ‘new normal’ [32, 33]. When the direct effects have improved, for example after traumatic brain injury or post‐COVID infection, the internal model is not updated back to a more normal state and continues to respond as if the acute trigger is still present (Figure 2). In other words, there is a failure of habituation to the acute trigger. The symptoms are typically amplified through self‐monitoring, hypervigilance, anticipatory and performance anxiety [34], which in turn further reduce the amount of attentional resources available for information processing and task performance [4, 32, 35]. Other potential perpetuating factors include repeated investigations, time off work and litigation [15, 32, 33, 34].

Our study is a preliminary investigation, and we acknowledge it has limitations. The information obtained is limited to what was registered in the clinical letters, and recall bias is a possibility, including chances of overreporting of physical events close to the onset of functional symptoms, and underreporting of significant life events [36]. The retrospective nature of this study makes it difficult to ascertain whether the comorbidities arose before or after the precipitating events for all cases. We selected patients with at least 1 year of follow‐up to increase confidence in the diagnosis of FCD, so it is possible that some patients might not come to medical attention if they experience a self‐limited remitting illness, and we cannot exclude that some patients may have an underlying neurodegeneration like Alzheimer's disease. In addition, FCD post‐acute precipitating events may present to other settings including TIA, dizziness and long‐COVID dedicated clinics. Finally, although we recorded patients with a formal diagnosis of anxiety and/or depression, patients with FCD frequently have subclinical psychiatric symptomatology which may further impact symptom development and management.

## 5 Conclusion

In conclusion, this study may provide further insight into the mechanisms underlying FCD and open future avenues for better disease management and personalised treatment strategies [37]. Future research may explore whether precipitating events aid in the development of better diagnostic criteria and preventative measures. Longitudinal studies are also needed to study factors related to persistence of cognitive difficulties in patients with acute onset of FCD.

## Author Contributions

**Veronica Cabreira:** data curation, formal analysis, validation, methodology, writing – original draft, investigation, conceptualization. **Laura McWhirter:** writing – review and editing, data curation. **Jon Stone:** methodology, writing – review and editing, supervision, funding acquisition, validation. **Alan Carson:** funding acquisition, writing – review and editing, supervision, data curation, validation.

## Conflicts of Interest

The authors declare no conflicts of interest.

## Data Availability

The data that support the findings of this study are available upon request from the corresponding author. The data are not publicly available due to privacy or ethical restrictions.
